# 4-HNE-induced cellular dysfunction from lipid peroxidation: a potential therapeutic target in diabetic cardiomyopathy

**DOI:** 10.3389/fcell.2025.1663094

**Published:** 2026-01-02

**Authors:** Nan Jiang, Yanchun Ma, Huijun Chen, Chengjia Li

**Affiliations:** 1 Geriatric Psychiatry, Second People’s Hospital of Zhoushan, Zhoushan, China; 2 Academic Research Department, Heilongjiang University of Chinese Medicine, Harbin, China; 3 Heilongjiang University of Chinese Medicine affiliated second hospital, Harbin, China; 4 Heilongjiang University of Chinese Medicine, Harbin, China

**Keywords:** diabetic cardiomyopathy, 4-HNE, oxidative stress, lipid peroxidation, organelles

## Abstract

Diabetic cardiomyopathy (DCM) is one of the crucial causes leading to heart failure and adverse outcomes in patients with diabetes mellitus; however, effective strategies targeting its molecular pathological mechanisms and therapies are currently lacking. DCM is primarily characterized by early diastolic dysfunction, cardiomyocyte apoptosis, and fibrosis. Its disease progression is relatively insidious, eventually evolving into heart failure with preserved ejection fraction. The intrinsic metabolic environment of diabetes markedly exacerbates oxidative stress, and the accumulated polyunsaturated fatty acids within cardiomyocytes are highly susceptible to lipid peroxidation, leading to the excessive generation of 4-hydroxy-2-nonenal (4-HNE). The pivotal role of this reactive aldehyde in promoting the progression of DCM has been extensively demonstrated in animal, cellular, and clinical models. However, its subcellular targets and the underlying molecular mechanisms remain inadequately elucidated. Organelles, as central executors of diverse intracellular functions, may serve as potential sites of 4-HNE-induced interference and therapeutic targeting. This article focuses on the central role of 4-HNE in triggering energy depletion, calcium overload, autophagic flux blockade, and ferroptosis through its interactions among mitochondria, endoplasmic reticulum, lysosomes, and other organelles. On the basis of existing evidence, potentially translatable therapeutic avenues include ALDH2 activators, G protein–coupled receptor 40 (GPR40) agonists, mitochondria-targeted antioxidants and ferroptosis inhibitors. The aim is to provide a theoretical foundation and reference for the clinical identification of myocardial injury in DCM, model replication, and the development of targeted intervention strategies.

## 1 Introduction

Diabetes mellitus, a global chronic metabolic disorder, continues to rise in both incidence and mortality and is now a major contributor to the growing burden of cardiovascular diseases. In recent years, the prevalence of diabetes mellitus (DM) has increased markedly alongside global population aging. The International Diabetes Federation (IDF) projects that the global number of people with diabetes will reach 693 million by 2045 (Cho et al., 2018). Diabetes-related cardiovascular complications are directly linked to over half of all deaths among individuals with diabetes (Ma et al., 2022). The cardiovascular impact of diabetes extends beyond traditional coronary artery disease. Increasing evidence suggests that diabetes can directly impair cardiomyocytes through chronic metabolic dysregulation and oxidative stress (Peng et al., 2023), even in the absence of coronary artery disease or hypertension, ultimately leading to a distinct cardiomyopathy known as diabetic cardiomyopathy (DCM) (Einarson et al., 2018). DCM was first described by Rubler in 1972 and is defined as a distinct myocardial disorder that occurs independently of hypertension and coronary artery disease. Its primary etiology is myocardial injury caused by hyperglycemia, rather than by hemodynamic overload. DCM is primarily characterized by early diastolic dysfunction, cardiomyocyte apoptosis, and myocardial fibrosis, and may gradually progress to heart failure with preserved ejection fraction (HFpEF) (Ozturk et al., 2021). Its progression is strongly associated with the duration of diabetes and the degree of glycemic control (Dunlay et al., 2019). A major concern is that the onset of DCM is typically insidious and gradual, with few or no specific symptoms in its early stages. As a result, it is often not diagnosed until the disease has advanced to the clinical stage or manifest heart failure.

## 2 Imbalanced antioxidant processes in DCM

The intrinsic metabolic milieu of DCM, characterized by hyperglycemia and elevated free fatty acids, markedly exacerbates oxidative stress. In response to excessive reactive oxygen species (ROS), cells activate a complex antioxidant defense system. Enzymatic antioxidants include superoxide dismutase (SOD), catalase (CAT), and glutathione peroxidase (GPX), while non-enzymatic antioxidants consist of small molecules such as vitamin C, vitamin E, and glutathione (GSH). These components act synergistically to neutralize ROS and halt free radical chain reactions (Xiao Liang, 2024). Once cellular antioxidant defenses are depleted, hyperglycemia drives excessive mitochondrial ROS production and promotes protein glycation, leading to the formation of advanced glycation end products (AGEs). These events further exacerbate oxidative stress and trigger inflammatory responses. Concurrently, under lipotoxic conditions, accumulated polyunsaturated fatty acids in cardiomyocytes become preferential targets of ROS, rendering them highly vulnerable to lipid peroxidation (LPO).

LPO is among the earliest and most sustained forms of cellular damage in DCM (Bhatti et al., 2022). Cardiomyocytes, owing to their intense metabolic rate, elevated energy consumption, and mitochondrial richness, are exceptionally prone to organelle dysfunction under oxidative conditions (Gianazza et al., 2021). Growing evidence has identified LPO as a critical trigger of ferroptosis, especially under circumstances of GSH depletion, GPX4 dysfunction, and excess polyunsaturated fatty acids (PUFAs) (Chen et al., 2024). LPO can trigger irreversible cell death by inflicting permanent structural disruption of membrane lipids (Ding et al., 2024; Jin et al., 2022). Emerging evidence indicates that LPO exerts functions beyond its role in ferroptosis. It is not simply a terminal outcome of cell death but also acts as an upstream trigger of complex subcellular injury. Sustained disruption of inter-organelle communication constitutes a core pathological mechanism underlying cellular stress, remodeling processes, and loss of function. This coordinated organelle dysfunction disturbs cardiomyocyte metabolic regulation, energy balance, inflammatory signaling, and structural stability, thereby accelerating the transition of diabetic cardiomyopathy from early metabolic imbalance to irreversible tissue remodeling (Ritchie and Abel, 2020; Marwick et al., 2018).

Among LPO-derived aldehydes, 4-hydroxynonenal (4-HNE) is considered the most cytotoxic. In addition to its extensive role in protein adduction and inflammatory signaling, it also serves as a widely recognized biomarker of oxidative stress (Li Y. et al., 2022; Chaudhary et al., 1994). In recent years, 4-HNE has emerged as a research focus, chiefly because it arises from the peroxidation of ω-6 polyunsaturated fatty acids and, under hyperglycemic/lipotoxic conditions, exhibits higher steady-state levels and cumulative burden by providing a more faithful readout of myocardial endogenous lipid peroxidation intensity (Esterbauer et al., 1991; Ayala et al., 2014). Second, as a moderately reactive α,β-unsaturated aldehyde, 4-HNE has a relatively longer half-life and the capacity to diffuse across membranes; unlike hyper-reactive aldehydes that react locally, it more readily spreads between organelles and drives system-wide injury (Uchida, 2003; Schaur et al., 2015). In addition, 4-HNE undergoes selective Michael addition and Schiff-base reactions with Cys/His/Lys side chains, generating persistent protein adducts that perturb key metabolic enzymes and ion-handling proteins, thereby amplifying pathology. Although cells possess detoxification routes (e.g., ALDH2, AKR, GSTA4), these pathways often become saturated or imbalanced under stress, facilitating pathological 4-HNE accumulation (Doorn and Petersen, 2003; Srivastava et al., 2002; Awasthi et al., 2003). Importantly, 4-HNE and its adducts are reproducibly and quantitatively measurable, making it a tractable biomarker with translational potential (Perluigi et al., 2012; Weber et al., 2013). Current evidence indicates that, across multiple models, 4-HNE activates inflammasomes, promotes calcium overload, impairs autophagy–lysosome flux, and interferes with transcriptional regulation and mRNA stability by modifying nuclear or RNA-binding proteins (Xiao Liang, 2024; Zeng et al., 2024).

## 3 Structural characteristics of 4-HNE

4-HNE is a nine-carbon α,β-unsaturated aldehyde (C_9_H_16_O_2_) generated during lipid peroxidation (Jin et al., 2023). Structurally, it contains a conjugated carbonyl–double bond system and a hydroxyl group at the 4-position. At room temperature, 4-HNE is a colorless, oily compound with moderate lipophilicity, facilitating its intracellular diffusion. Owing to its electrophilic α,β-unsaturated carbonyl structure, 4-HNE is a prototypical Michael acceptor that readily forms covalent adducts with biomolecules. It preferentially reacts with nucleophilic residues in proteins—such as cysteine, lysine, and histidine—via Michael addition, and can also form Schiff bases with lysine ε-amino groups (Li Y. et al., 2022). These reactions result in irreversible modifications of proteins and nucleic acids, altering their structure and function. Furthermore, its hydrophobicity enables membrane intercalation and disruption of lipid components. Compared with related aldehydes like acrolein, the hydroxyl moiety of 4-HNE imparts slightly higher polarity, allowing it to exert effects in both membrane-associated and cytosolic contexts.

4-HNE is one of the most prominent and well-characterized lipid peroxidation products, distinguishable from other aldehydes such as malondialdehyde (MDA) and acrolein (Pizzimenti et al., 2013; Moghe et al., 2015). MDA, a three-carbon dialdehyde, arises as a low-molecular-weight byproduct of extensive peroxidative fragmentation of polyunsaturated fatty acids. While it is widely employed as a biomarker of oxidative injury, its electrophilic reactivity is markedly lower than that of 4-HNE. MDA primarily exerts its genotoxic effects by forming adducts with DNA bases, leading to mutagenesis (Li Y. et al., 2022). In contrast, 4-HNE, characterized by a conjugated aldehyde structure, exhibits high reactivity. It can broadly modify proteins, disrupt intracellular signaling, and is considered the most cytotoxic lipid peroxidation product (Ursini and Maiorino, 2020). Acrolein, the smallest α,β-unsaturated aldehyde and lacking the hydroxyl group found in 4-HNE, is the most electrophilic of the three. It rapidly reacts with nucleophilic molecules such as glutathione, leading to depletion of cellular antioxidants; as a result, its physiological concentration remains relatively low *in vivo* (Stevens and Maier, 2008). Overall, although acrolein possesses the highest reactivity, its overall intracellular abundance is lower than that of MDA and 4-HNE (Esterbauer et al., 1991). Under pathological conditions characterized by both high abundance and high toxicity, 4-HNE often exerts long-lasting effects on protein function and cellular signaling pathways (Schaur et al., 2015). In contrast, MDA, due to its ease of detection and high sensitivity, is more commonly used as an indicator of oxidative damage, while acrolein contributes more directly to acute cytotoxicity (Ayala et al., 2014). It is worth noting that at low concentrations, 4-HNE has been found to function as an intracellular signaling molecule, participating in the regulation of processes such as transcription, proliferation, and differentiation. However, at higher concentrations, its deleterious effects predominate (Singhal et al., 2015). Due to its unique structure, 4-HNE stands out among lipid peroxidation products for its intermediate molecular size and high bioactivity, making it a focal point of interest in chronic pathological conditions such as diabetes (Dham et al., 2021) (Table 1).

**TABLE 1:** Differences between 4-HNE, MDA and acrolein.

LPO products	Chemical formula	Chemical structure diagram	Key features	Physiological range (free, μM)
4-HNE	C9H16O2	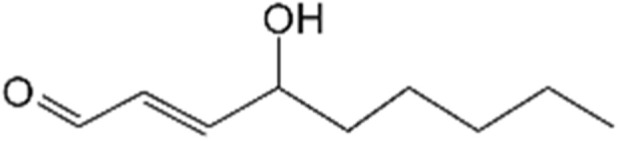	Medium molecular weight, high activity, sustainable protein modification	<0.15
MDA	C3H4O2	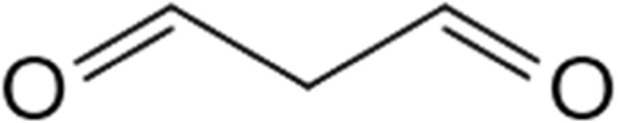	Small molecule dialdehyde, low activity, often used as an indicator of oxidative damage	<0.10
Acrolein	C3H4O	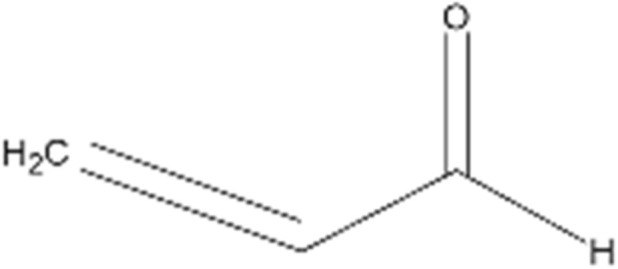	Strongest electrophile, low production, causes acute toxicity	<0.50

## 4 Formation process of 4-HNE in DCM

Extensive research demonstrates that 4-HNE is predominantly generated through the peroxidative breakdown of ω-6 PUFA embedded in membrane phospholipids (Bose et al., 2021). In non-enzymatic, free radical–driven lipid peroxidation, ROS initially abstract bis-allylic hydrogen atoms from substrates such as linoleic and arachidonic acids, initiating lipid radical formation. These radicals subsequently react with molecular oxygen to form lipid peroxyl species, which propagate the chain reaction by extracting hydrogen atoms from adjacent fatty acyl chains, producing lipid hydroperoxides (LOOH). This reaction sequence sustains a self-amplifying radical cascade. In the presence of transition metals, LOOH undergo Hock rearrangement and C–C bond cleavage, yielding aldehydic fragments—including saturated, α,β-unsaturated, and γ-unsaturated aldehydes—with 4-HNE being a major product. Under diabetic conditions, elevated ROS levels readily trigger these spontaneous peroxidation cascades in membrane lipids, leading to significant intracellular accumulation of 4-HNE in cardiomyocytes.

In addition to non-enzymatic free radical–driven mechanisms, intracellular lipid-oxidizing enzymes also contribute to 4-HNE formation. Within the pro-inflammatory and oxidative microenvironment of DCM, upregulated lipoxygenase (LOX) and cyclooxygenase (COX) pathways promote enzymatic generation of 4-HNE. Specifically, 15-LOX catalyzes the oxidation of arachidonic and linoleic acids to produce hydroperoxides such as 15-hydroperoxyeicosatetraenoic acid (15-HpETE) and 13-hydroperoxyoctadecadienoic acid (13-HpODE), which subsequently degrade into 4-HNE. Thus, in diabetic myocardium, both enzymatic and non-enzymatic oxidative processes act synergistically to drive excessive 4-HNE accumulation. When diabetes impairs cardiac antioxidant enzyme activity, this peroxidative burden is further amplified.

## 5 The role of 4-HNE in DCM cardiomyopathy

A substantial body of animal research has demonstrated a pivotal role for 4-HNE in driving myocardial remodeling in DCM, highlighting its significance in the pathogenesis of diabetic cardiomyopathy. Xiong et al. developed a streptozotocin (STZ)-induced diabetic mouse model and found that myocardial 4-HNE levels were significantly elevated. Administration of the mitochondrial-targeted antioxidant SS-31 or the ferroptosis inhibitor ferrostatin-1 (Fer-1) markedly suppressed 4-HNE accumulation and improved cardiac function, as reflected by enhanced ejection fraction (EF) and fractional shortening (FS). These findings suggest that elevated 4-HNE in diabetes mediates lipid peroxidation–driven myocardial injury and worsens cardiac dysfunction. Targeting mitochondrial oxidative stress effectively lowers 4-HNE levels and preserves cardiac performance (Xiong et al., 2024). Roy et al. explored the role of 4-HNE in a diabetic heart failure model with preserved ejection fraction (HFpEF). In db/db mice, elevated myocardial 4-HNE levels were linked to coronary microvascular rarefaction and deteriorated cardiac performance. The study revealed that 4-HNE induces myocardial adiponectin (APN) resistance, characterized by increased systemic APN concentrations but diminished myocardial APN signaling, thereby impairing coronary angiogenesis. Activation of mitochondrial aldehyde dehydrogenase 2 (ALDH2) promoted 4-HNE detoxification, restored APN signaling and vascular regeneration, and ultimately improved diastolic function in diabetic hearts (Roy et al., 2022). Another study investigating the effects of 4-HNE on cardiomyocyte DNA repair capacity reported that the activity of 8-oxoguanine DNA glycosylase 1 (OGG1), a key enzyme responsible for repairing oxidative DNA damage, was significantly reduced in the myocardium of db/db mice, accompanied by elevated 4-HNE adduct levels. Impaired ALDH2 activity in diabetic hearts may promote 4-HNE accumulation, which subsequently binds to and compromises OGG1 function. Moreover, exogenous 4-HNE was found to directly modify and inhibit OGG1 activity. These findings suggest that 4-HNE covalently modifies essential DNA repair enzymes, thereby diminishing the DNA repair capacity of cardiomyocytes and potentially worsening cellular injury and functional deterioration (Pan et al., 2020). In an STZ-induced diabetic cardiomyopathy model, Zhang et al. demonstrated that overexpression of fibroblast growth factor 21 (FGF21) markedly reduced myocardial 4-HNE accumulation. In contrast, untreated diabetic mice exhibited pronounced cardiac hypertrophy, fibrosis, and concurrent accumulation of iron and 4-HNE—pathological features that were substantially attenuated in the FGF21-overexpressing group. Conversely, silencing FGF21 aggravated ferroptosis-related changes in the diabetic myocardium, including further increases in 4-HNE levels. These results suggest that the cardioprotective effect of FGF21 in DCM may involve suppression of lipid peroxidation and excessive 4-HNE production, thereby preserving myocardial architecture and function (Wang et al., 2024). Lee et al. reported that the novel aldehyde dehydrogenase 2 (ALDH2) activator, AD 9308, significantly enhanced both systolic and diastolic cardiac function in STZ-induced diabetic mice. AD 9308 reduced serum 4-HNE levels and myocardial 4-HNE–protein adducts in a dose-dependent manner. As a result, myocardial fibrosis, inflammation, and apoptosis were markedly attenuated, accompanied by improvements in mitochondrial function and calcium homeostasis (Lee et al., 2021).

At the *in vitro* cellular level, numerous studies have employed high-glucose or high-lipid-induced cardiomyocyte models to examine how 4-HNE dysregulation affects cardiomyocyte function, further substantiating its pathogenic role in DCM. In one study, H9c2 cells exposed to 35 mM glucose for 24 h exhibited significantly increased intracellular 4-HNE and MDA levels compared to normoglycemic controls, accompanied by oxidative stress. Co-treatment with the ferroptosis inhibitor ferrostatin-1 (Fer-1) or the mitochondrial-targeted antioxidant SS-31 markedly suppressed high glucose–induced accumulation of 4-HNE and MDA, thereby mitigating lipid peroxidation–driven cellular injury (Xiong et al., 2024). Yun et al. exposed H9c2 cardiomyocytes to the saturated fatty acid palmitic acid (PA) and observed a significant elevation in intracellular superoxide anions and 4-HNE levels. Treatment with AM1638, a G protein–coupled receptor 40 (GPR40) agonist, markedly suppressed PA-induced overproduction of ROS and 4-HNE. However, pharmacological inhibition of the AMPK pathway abolished the antioxidant effect of AM1638. These findings indicate that lipid peroxidation products like 4-HNE contribute to high-fat–induced cardiomyocyte damage and that activation of the AMPK signaling pathway may serve as a protective mechanism against such oxidative stress (Yun et al., 2025). Zhai et al. exposed H9c2 cardiomyocytes to various concentrations of 4-HNE and observed that it significantly induced necroptosis in both a dose- and time-dependent manner. Knockdown of receptor-interacting protein kinase 1 (RIP1), a central mediator of necroptosis, effectively inhibited 4-HNE–induced cell death. These results indicate that 4-HNE promotes necroptotic signaling by disrupting ubiquitin-dependent degradation of RIP1, leading to its stabilization and activation of the necroptotic cascade (Zhai et al., 2021). Although primarily focused on ischemia–reperfusion injury, the study’s findings imply that excessive 4-HNE accumulation under high-glucose and high-lipid conditions may likewise initiate aberrant cardiomyocyte death, contributing to cell loss in DCM. Multiple *in vitro* studies have used 4-HNE as a key marker of oxidative stress–mediated injury. For example, cardiomyocytes deficient in the circadian clock gene Bmal1 display enhanced oxidative stress in high-glucose/high-lipid environments, likely accompanied by elevated 4-HNE levels, leading to mitochondrial dysfunction and cell damage (Kohsaka et al., 2014). Similarly, ALDH2 overexpression in high-glucose–exposed H9c2 cells has been shown to attenuate NLRP3 inflammasome activation and apoptosis, likely via enhanced detoxification of 4-HNE and related reactive aldehydes, thereby mitigating oxidative injury (Liu et al., 2018). Collectively, these studies reinforce the central role of 4-HNE as a key mediator of cellular stress responses triggered by high-glucose and high-lipid environments.

Clinical evidence further supports these findings. A 2024 study comparing myocardial tissue from healthy individuals and diabetic heart failure patients demonstrated significantly elevated levels of 4-HNE and its protein adducts in diabetic hearts, suggesting that the diabetic milieu exacerbates myocardial lipid peroxidation injury (Gawargi and Mishra, 2024). Dham et al. systematically reviewed clinical data and reported consistently elevated 4-HNE and 4-HNE–protein adduct levels across multiple organ systems in diabetic patients compared to healthy controls. They proposed that 4-HNE may serve as a promising biomarker for diabetes-related end-organ damage, including myocardial injury characteristic of diabetic cardiomyopathy (Dham et al., 2021). Toyokuni et al. reported that serum levels of 4-HNE–modified albumin were significantly elevated in patients with type 2 diabetes compared to healthy controls (736 pmol/mL vs. 611 pmol/mL), reflecting enhanced systemic lipid peroxidation associated with the diabetic condition (Toyokuni et al., 2000). Giuseppe et al. reported that 4-HNE accumulation impairs the differentiation of adipocyte precursor cells, thereby contributing to obesity-related disruptions in glucose homeostasis (Murdolo et al., 2023). These findings imply that poor glycemic control or prolonged diabetes duration leads to greater oxidative damage in the myocardium. Consequently, circulating 4-HNE levels may increase, further impairing glucose regulation and perpetuating a vicious cycle of metabolic dysfunction (Jaganjac et al., 2013).

## 6 Organelle dysfunction induced by 4-HNE in DCM

**FIGURE 1:**
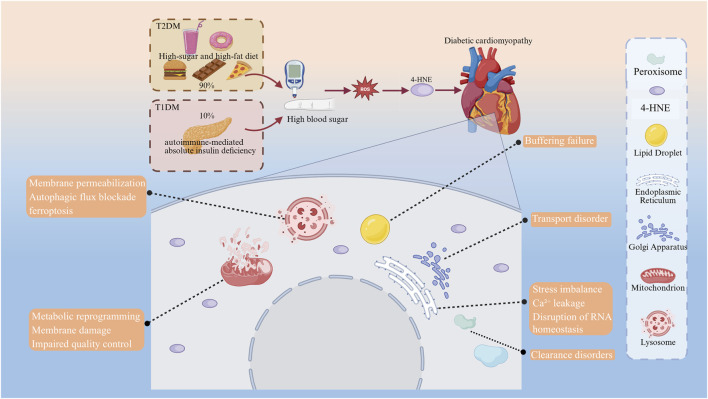
4-HNE causes organelle damage in DCM cardiomyocytes.

In summary, extensive animal, cellular, and clinical studies have established that 4-HNE, a terminal product of lipid peroxidation, plays a pivotal pathogenic role in the progression of diabetic cardiomyopathy. Given that organelles orchestrate essential intracellular processes, further elucidation of how 4-HNE impairs organelle function in cardiomyocytes may yield novel mechanistic insights and advance therapeutic strategies. Previous studies have shown that mitochondria and lysosomes are especially vulnerable to 4-HNE–induced injury (Yamashima, 2024; Du et al., 2022). Moreover, 4-HNE may further accelerate the progression of diabetic cardiomyopathy by impairing additional organelles—including the endoplasmic reticulum, Golgi apparatus, lipid droplets, and peroxisomes—through interference with protein folding, post-translational modification, and lipid metabolic processes (Gallo et al., 2020). Given the essential roles of these organelles in oxidative phosphorylation, protein synthesis and degradation, and lipid storage and turnover, elucidating how 4-HNE disrupts their functions will be the focus of the following discussion (Figure 1).

### 6.1 Mitochondria

**FIGURE 2:**
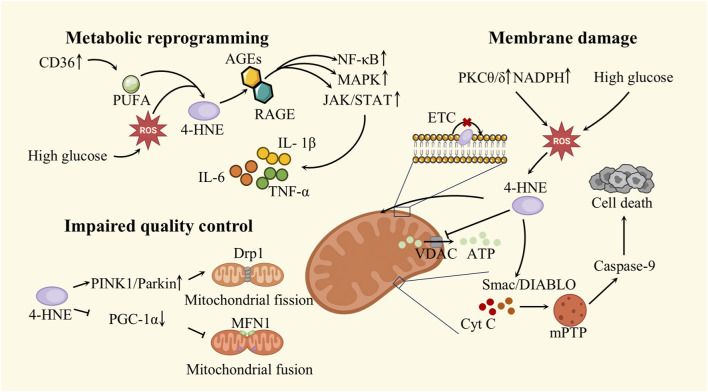
Damage caused by 4-HNE in mitochondria.

As one of the most metabolically active terminally differentiated cell types, cardiomyocytes depend heavily on mitochondria to sustain continuous ATP generation via oxidative phosphorylation. In these cells, mitochondria account for over 30% of cytoplasmic volume and contribute approximately 8% of the body’s total ATP output (Bisaccia et al., 2021). In diabetic rats, myocardial mitochondrial capacity for steady-state ATP synthesis decreases by nearly one-third, accompanied by a ∼12% decline in oxygen utilization efficiency. Under hyperglycemia, a shift from glucose toward fatty-acid oxidation lowers oxygen efficiencyand heightens mitochondrial electron leak, thereby increasing 4-HNE generation. The ensuing 4-HNE adducts on membrane proteins and cardiolipin deepen mitochondrial depolarization, amplify proton leak, and blunt coupling efficiency. In parallel, 4-HNE disrupts the fusion–fission balance and impairs mitophagy, derailing mitochondrial quality control and permitting the accumulation of dysfunctional organelles—reinforcing a self-propagating loop of oxidative stress, energetic insufficiency, and structural remodeling. (Pham et al., 2014) (Figure 2).

#### 6.1.1 Metabolic reprogramming

In DCM, cardiomyocytes experience significant alterations in mitochondrial metabolic pathways. Due to the myocardium’s high reliance on a continuous energy supply, it is especially susceptible to mitochondrial dysfunction (Liu et al., 2025). Insulin resistance significantly impairs glucose uptake and oxidation, thereby reducing the contribution of glycolysis and glucose oxidation to ATP production (Lee et al., 2022). Concurrently, the upregulation of the fatty acid transporter CD36 enhances lipid substrate uptake and drives compensatory β-oxidation in cardiomyocytes. While this metabolic reprogramming temporarily maintains energy homeostasis, it also results in the accumulation of PUFAs and lipotoxic intermediates, which serve as precursors for 4-HNE generation. Elevated 4-HNE levels can activate the AGEs–RAGE signaling pathway, thereby exacerbating chronic inflammation (Yang et al., 2016). AGEs are terminal compounds generated by non-enzymatic reactions between reducing sugars and amino groups on proteins, lipids, or nucleic acids (Twarda-Clapa et al., 2022). Their accumulation is significantly accelerated under hyperglycemic conditions (Takeuchi et al., 2021). AGEs promote oxidative stress by generating free radicals through interactions with transition metals such as iron and copper and by impairing antioxidant enzyme systems, thereby diminishing the cellular ability to eliminate ROS (Wei et al., 2013). The receptor for AGEs (RAGE), their principal binding partner, is abundantly expressed on cardiomyocytes, endothelial cells, and immune cells (Hudson and Lippman, 2018). Under diabetic conditions, AGEs–RAGE interactions activate downstream signaling pathways including NF-κB, MAPK, and JAK/STAT (Shen et al., 2020), triggering the release of pro-inflammatory cytokines such as IL-6, IL-1β, and TNF-α (Moldogazieva et al., 2019), which collectively contribute to myocardial injury and structural remodeling.

#### 6.1.2 Membrane damage

Mitochondrial membranes, due to their lipid-rich composition, are highly vulnerable to ROS attack in diabetic cardiomyopathy, which in turn enhances the toxicity of 4-HNE. The activation of protein kinase C isoforms θ and δ (PKCθ/δ), along with the upregulation of NADPH oxidase, further enhances ROS production (Geraldes and King, 2010). Due to their high susceptibility to oxidative modification, PUFAs become central substrates in the LPO cascade.

Due to its hydrophobic nature, 4-HNE readily integrates into the mitochondrial phospholipid bilayer, disrupting lipid organization and increasing membrane permeability (Santin et al., 2020). This leads to ionic gradient collapse and loss of membrane integrity. The α,β-unsaturated aldehyde group of 4-HNE enables it to form covalent adducts with thiol or amino residues on key mitochondrial proteins such as adenine nucleotide translocase (ANT) and voltage-dependent anion channel (VDAC), thereby impairing ATP/ADP exchange and ion flux regulation (Jaganjac et al., 2020; Yarian et al., 2005). These combined insults contribute to mitochondrial membrane potential depolarization. Under the synergistic stress of ROS accumulation and Ca^2+^ overload, this condition eventually induces sustained opening of the mitochondrial permeability transition pore (mPTP) (Kent et al., 2021).

Pathological opening of the mitochondrial permeability transition pore (mPTP) promotes the release of pro-apoptotic molecules such as cytochrome c (Cyt C) and Smac/DIABLO into the cytosol, thereby initiating the caspase-9–mediated intrinsic apoptotic cascade and triggering cardiomyocyte death (Zorov et al., 2014). At this point, 4-HNE functions not merely as a byproduct of oxidative stress but as a pathogenic effector that actively promotes cardiomyocyte apoptosis.

Notably, studies have demonstrated that in diabetic conditions, depletion of the mitochondrial thioredoxin-2 (Trx2)/GSH system in db/db mice disrupts redox homeostasis, resulting in lipid peroxidation of the mitochondrial membrane and impaired activity of respiratory chain complexes. Exogenous GSH supplementation restores mitochondrial respiration and attenuates myocardial injury associated with diabetes (Tocchetti et al., 2012).

#### 6.1.3 Impaired quality control

Persistent LPO–induced damage ultimately impairs mitochondrial quality control mechanisms. Mitochondrial dynamics—governed by the balanced interplay of fusion and fission—are crucial for sustaining cellular energy homeostasis and facilitating adaptive responses to stress (Zong et al., 2024). During mitochondrial dysfunction or elevated energy demand, fission promotes the removal of damaged fragments and the proliferation of intact mitochondria to maintain ATP output and compartmentalize injury. Conversely, fusion enables the mixing of mitochondrial contents, enhances substrate exchange, and preserves respiratory efficiency and membrane potential, thereby supporting mitochondrial integrity and metabolic adaptability (Pfluger et al., 2015). Dynamin-related protein 1 (Drp1), a cytoplasmic GTPase essential for mitochondrial fission, is activated by cellular stress or division signals. Once activated, Drp1 translocates to the mitochondrial outer membrane, where it assembles with adaptor proteins—such as FIS1, MFF, MID49, and MID51—to form a GTP-driven contractile ring that mediates mitochondrial scission (Pfluger et al., 2015). In contrast, mitofusin 1 (MFN1), a highly conserved outer membrane GTPase, regulates mitochondrial fusion by initiating membrane tethering, docking, and merging between adjacent mitochondria. Compared with its homolog MFN2, MFN1 exhibits higher GTPase activity and relies more extensively on GTP hydrolysis to facilitate membrane fusion (Chen et al., 2003).

Under 4-HNE–induced lipid peroxidation, mitochondrial dynamics shift toward excessive fission. This imbalance is marked by Drp1 hyperphosphorylation at Ser616, which amplifies fission activity, whereas suppressed MFN2 expression and aberrant OPA1 processing impair fusion, resulting in widespread mitochondrial fragmentation (Massaad and Klann, 2011; Patten et al., 2014). Simultaneously, aberrant activation of PINK1/Parkin-mediated mitophagy accelerates the loss of functional mitochondria (Yang et al., 2024). In parallel, downregulation of PGC-1α and inhibition of the NRF1/TFAM pathway reduce mitochondrial DNA (mtDNA) copy number and impair biogenesis, limiting the replenishment of damaged mitochondria (Cioffi et al., 2022). Collectively, these alterations form a pathogenic axis whereby 4-HNE–driven mitochondrial dysfunction culminates in cardiomyocyte death and myocardial remodeling, thus contributing to the progression of diabetic cardiomyopathy.

### 6.2 Endoplasmic reticulum

The endoplasmic reticulum (ER) is a highly specialized organelle in eukaryotic cells that performs essential physiological functions, including lipid metabolism, calcium homeostasis, and the folding and maturation of secretory and membrane proteins (Wang et al., 2018). Most newly synthesized membrane-bound and secreted proteins are co-translationally translocated into the ER lumen, where they undergo critical post-translational modifications and proper folding to ensure correct trafficking and functionality.

**FIGURE 3:**
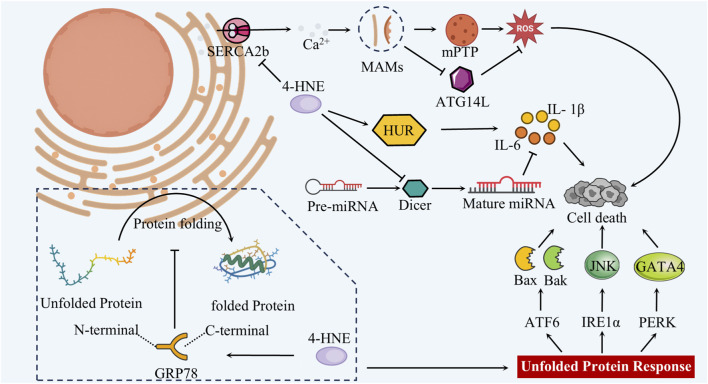
Damage caused by 4-HNE in the endoplasmic reticulum.

Oxidative stressors such as 4-HNE disrupt ER homeostasis by promoting the accumulation of misfolded or unfolded proteins within the ER lumen, thereby impairing its internal environment (Ljubkovic et al., 2019; Yang et al., 2015; Sun et al., 2019). When this stress is sustained or surpasses the adaptive threshold, the unfolded protein response (UPR) transitions from a protective mechanism to a pro-apoptotic pathway, triggering programmed cell death to eliminate irreparably damaged cells (Xu et al., 2012; Guo et al., 2015) (Figure 3).

#### 6.2.1 Stress imbalance

To sustain its highly organized protein synthesis and processing functions, the ER lumen maintains elevated calcium levels and oxidative redox conditions, and is enriched with calcium-dependent molecular chaperones such as GRP78 and GRP94 to ensure proper protein folding (Wang et al., 2018). Under oxidative stress, 4-HNE covalently modifies ER membrane proteins and luminal chaperones, disrupting their structure and activity, thereby impairing protein folding (Negre-Salvayre et al., 2010). GRP78, a central sensor of the UPR, binds unfolded proteins via its N-terminal domain and interacts with UPR signaling branches through its C-terminal domain, maintaining these pathways in an inactive state under basal conditions (Ibrahim et al., 2019). 4-HNE can modify critical residues of GRP78, impairing its chaperone activity (Liang et al., 2025). This disruption hinders GRP78’s ability to recognize misfolded proteins, compromising ER protein quality control and triggering UPR activation (Wu et al., 2015).

In its initial compensatory phase, the UPR alleviates ER stress by suppressing global protein synthesis, upregulating chaperone levels, and facilitating the degradation of misfolded proteins. However, prolonged exposure to high glucose and lipids drives the UPR toward pathological activation through three canonical signaling pathways: PERK, IRE1α, and ATF6 (Liu X. et al., 2024). The PERK axis promotes CHOP expression, which upregulates the pro-apoptotic proteins Bax and Bak, increases mitochondrial membrane permeability, and triggers apoptosis (Ma et al., 2020). The IRE1α pathway activates JNK signaling, promoting inflammatory cytokine production and exacerbating insulin resistance (Su et al., 2013). Although ATF6 initially supports ER homeostasis, sustained activation upregulates GATA4, contributing to myocardial hypertrophy and structural remodeling (Mao et al., 2006). Chronic dysregulation of these UPR pathways leads to enhanced cardiomyocyte apoptosis, inflammation, and structural deterioration, forming a key mechanism underlying ER dysfunction in diabetic cardiomyopathy (Liu et al., 2017).

#### 6.2.2 Ca^2+^ leakage

The ER functions as the main intracellular calcium reservoir. Due to its membrane’s high content of polyunsaturated fatty acids (Szymański et al., 2017), it is highly vulnerable to 4-HNE–induced lipid peroxidation. Once embedded in the ER phospholipid bilayer, 4-HNE disrupts membrane integrity and inhibits sarco/endoplasmic reticulum Ca^2+^-ATPase 2b (SERCA2b) activity, leading to excessive Ca^2+^ leakage from the ER lumen (Tashkandi et al., 2025).

Dysregulated calcium efflux impairs the calcium-dependent environment necessary for molecular chaperone–mediated protein folding and exacerbates mitochondrial dysfunction via inter-organelle crosstalk. Free Ca^2+^ is rapidly transferred to mitochondria through mitochondria-associated membranes (MAMs), specialized contact sites between the ER and mitochondria (Liu Y. et al., 2024). This calcium influx induces mitochondrial Ca^2+^ overload and ROS bursts, promoting abnormal opening of the mitochondrial permeability transition pore (mPTP) and amplifying the lipid peroxidation cascade (Yang et al., 2015).

Furthermore, disruption of MAM integrity impairs the recruitment of autophagy-initiating proteins such as ATG14L, thereby hindering the clearance of damaged ER and mitochondria and exacerbating intracellular oxidative stress (Li Y. E. et al., 2022). Overall, these changes suggest that 4-HNE not only disrupts ER integrity but also induces mitochondrial dysfunction via Ca^2+^ imbalance, forming a pathological inter-organelle positive feedback loop that accelerates the progression of DCM.

#### 6.2.3 Disruption of RNA homeostasis

4-HNE also disrupts the protein-folding and quality control functions of the ER by altering RNA homeostasis and modulating extracellular vesicle signaling (Yang et al., 2015). Human antigen R (HuR), an mRNA-binding protein, enhances the stability of inflammatory cytokine transcripts such as IL-6 and IL-1β by prolonging their half-lives and promoting their translation. Although direct aldehyde modification of HuR by 4-HNE has not been definitively confirmed, multiple studies have shown that 4-HNE–induced oxidative stress activates signaling pathways that regulate HuR phosphorylation and dephosphorylation, significantly increasing its binding affinity for inflammatory mRNAs. Within this 4-HNE–enriched microenvironment, HuR-mediated transcript stabilization contributes to amplified local myocardial inflammation (Suresh Babu et al., 2015).

Additionally, 4-HNE disrupts the microRNA (miRNA) processing pathway, thereby impairing post-transcriptional gene silencing. It achieves this by covalently binding to and inhibiting Dicer, a critical RNase responsible for cleaving and loading precursor miRNAs into mature miRNAs. Inhibition of Dicer activity results in the derepression of inflammatory genes, amplifying their expression. Studies have shown that elevated myocardial 4-HNE levels in heart failure patients are strongly correlated with reduced Dicer function and impaired miRNA biogenesis. Notably, pharmacological activation of ALDH2 to detoxify 4-HNE restores Dicer activity and promotes normal miRNA maturation (Kiyuna et al., 2023).

### 6.3 Lysosome

**FIGURE 4:**
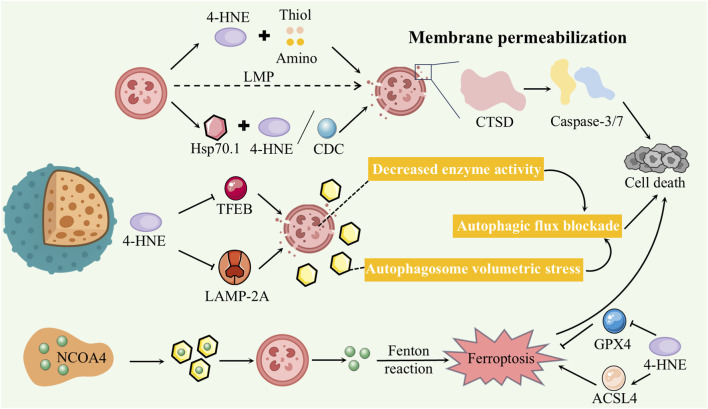
Damage caused by 4-HNE in lysosomes.

Lysosomes are vital organelles responsible for degrading intracellular waste and digesting autophagic cargo, with their membrane integrity being essential for maintaining proper autophagic flux. In DCM, lysosomal dysfunction is commonly observed, including increased membrane permeability, a decreased number of acidic lysosomes, and impaired autophagosome–lysosome fusion efficiency (Kobayashi et al., 2023) (Figure 4).

#### 6.3.1 Membrane permeabilization

As a highly reactive aldehyde, 4-HNE can directly target lysosomal membrane lipids and proteins by forming covalent adducts with thiol, amino, and other nucleophilic groups, thereby reducing membrane fluidity and compromising structural integrity—a process referred to as lysosomal membrane permeabilization (LMP) (Kobayashi et al., 2020). Heat shock protein 70.1 (HSP70.1), a molecular chaperone essential for maintaining lysosomal membrane stability, is especially prone to 4-HNE–induced carbonylation. This modification facilitates its cleavage by calcium-dependent caspases (CDCs), resulting in inactivation and eventual lysosomal rupture.

Upon lysosomal membrane disruption, glycosylated membrane proteins such as LAMP1 and LAMP2 become exposed to the cytosol. Their carbohydrate chains are recognized by Galectin-3 (Gal3), which binds to the damaged regions and forms visible puncta (Saimoto et al., 2025; Cañeque et al., 2025), making Gal3 a widely accepted marker of lysosomal injury (Jia et al., 2020). Loss of membrane integrity allows cathepsin D (CTSD), a lysosomal aspartic protease, to leak into the neutral cytoplasm (Kobayashi et al., 2020). Retaining enzymatic activity, CTSD cleaves essential cytosolic proteins and activates the mitochondrial apoptotic pathway via caspase-3/7 cascades, ultimately driving cardiomyocyte apoptosis (Kobayashi et al., 2017).

In cardiomyocytes exposed to high-glucose conditions, both the leakage and transcriptional upregulation of CTSD are markedly enhanced. Pharmacological blockade or genetic silencing of CTSD effectively attenuates high glucose–induced cell death, confirming its pathogenic role (Kobayashi et al., 2020). Therefore, 4-HNE–induced LMP is considered a central mechanism contributing to cardiomyocyte loss in diabetes (Yamashima et al., 2022).

#### 6.3.2 Autophagic flux blockade

4-HNE–induced lysosomal membrane injury profoundly impairs the autophagic process. Under normal physiological conditions, autophagosomes fuse with lysosomes, where their contents are efficiently degraded by acidic lysosomal hydrolases. In the context of DCM, however, compromised lysosomal acidification and diminished hydrolase activity impede the degradation of autophagosomes, resulting in their progressive accumulation (Kobayashi et al., 2023).

Various experimental models have demonstrated that in obesity and diabetes, cardiomyocytes show significant downregulation of lysosomal structural proteins and hydrolases. Notably, levels of LAMP2A are reduced, the proportion of acidic lysosomes declines, and autophagic substrates accumulate—collectively indicating a blockade of autophagic flux (Guan et al., 2023; Alcalai et al., 2021). Transcription factor EB (TFEB), the central regulator of lysosomal biogenesis and autophagy-related gene expression, is suppressed under high-glucose and high-lipid conditions. In both obese/diabetic mouse hearts and cardiomyocytes treated with high glucose and palmitate, TFEB protein levels are decreased and its nuclear translocation is impaired, leading to widespread disruption of the autophagy–lysosome pathway (Trivedi et al., 2016; Song et al., 2021).

Further investigations have revealed that excess saturated fatty acids, particularly palmitate, markedly reduce intracellular TFEB levels and inhibit autophagy (Trivedi et al., 2016; Trivedi et al., 2022). These findings suggest that in DCM, lipotoxicity resulting from altered substrate metabolism not only causes direct lysosomal injury through membrane peroxidation, but also impairs lysosomal renewal and autophagic capacity by downregulating critical regulators such as TFEB (Trivedi et al., 2016; Jaishy and Abel, 2016). The simultaneous suppression of TFEB and depletion of LAMP2A leads to a dual blockade of lysosomal biogenesis and autophagic flux. As a result, damaged proteins and organelles accumulate, triggering proteostasis stress, which further impairs cardiomyocyte function and accelerates the progression of DCM (Zheng et al., 2020; Arden et al., 2024).

#### 6.3.3 Ferroptosis

In addition to mediating terminal degradation in general autophagy, lysosomes play a key role in cellular iron homeostasis via NCOA4-dependent selective autophagy, termed ferritinophagy—recently recognized as a critical amplifier of LPO in DCM (Qin et al., 2021). During ferritinophagy, the autophagy receptor NCOA4 binds ferritin (Ferritin H/L) and delivers it to autophagosomes, which subsequently fuse with lysosomes for degradation and release of free Fe^2+^. This labile iron is then transported into the cytosol through DMT1 or TRPML1, where it catalyzes the Fenton reaction with H_2_O_2_, producing highly reactive hydroxyl radicals (·OH). These radicals initiate lipid radical chain reactions, promoting iron-dependent LPO and culminating in ferroptosis (Latunde-Dada, 2017; Lin et al., 2010).

Under DCM conditions, elevated 4-HNE levels have been shown to upregulate ACSL4—a key enzyme catalyzing PUFA esterification—and concurrently downregulate GPX4, the primary antioxidant enzyme responsible for reducing lipid hydroperoxides (Gawargi and Mishra, 2023). ACSL4 facilitates the incorporation of PUFAs into membrane phospholipids, rendering membranes more susceptible to oxidative attack, while reduced GPX4 activity weakens the cell’s defense against lipid peroxidation. This dysregulation leads to the uncontrolled buildup of iron-dependent lipid peroxides and activation of the ferroptosis pathway (Gawargi and Mishra, 2023). Overall, 4-HNE–induced LMP intensifies ferroptosis by promoting iron efflux, accelerating Fenton chemistry, and disrupting the ACSL4/GPX4 regulatory axis (Saimoto et al., 2025).

### 6.4 Other organelles

#### 6.4.1 Lipid droplet buffering failure

Lipid droplets (LDs) are indispensable organelles in cardiomyocytes that store neutral lipids and act as buffers to preserve energy homeostasis while mitigating lipotoxicity. Under normal conditions, excess free fatty acids (FFAs) are esterified into triglycerides (TGs), coated by structural proteins such as Perilipin-5 (PLIN5), and sequestered within LDs. These stored TGs are subsequently mobilized in a tightly regulated fashion to release FFAs for mitochondrial β-oxidation, thereby ensuring sustained energy production (Cui et al., 2022; Miner et al., 2023).

In the lipotoxic milieu of DCM, sustained overload of free fatty acids (FFAs) activates protein kinase A (PKA)–dependent phosphorylation of Perilipin-5 (PLIN5), triggering the release of the lipolytic complex adipose triglyceride lipase (ATGL) and its coactivator CGI-58. This event drives a rapid influx of FFAs into mitochondria, exacerbating ROS generation and facilitating the production of additional 4-HNE (Gallardo-Montejano et al., 2016). Consequently, the protective lipid droplet barrier is compromised, perpetuating a self-reinforcing cycle of excessive lipolysis and lipid peroxidation (Ayala et al., 2014).

Rab7, a small GTPase localized at the contact sites between LDs and lysosomes, undergoes phosphorylation at tyrosine 183 (Tyr183), enabling the recruitment of its effector protein RILP. RILP facilitates lysosomal docking and degradation of oxidatively damaged LD cores enriched with 4-HNE–laden triglycerides, thereby mitigating lipid peroxidation propagation (Ke et al., 2024). However, under prolonged high-glucose and high-lipid exposure, sustained activation of mechanistic target of rapamycin complex 1 (mTORC1) and depletion of the NAD^+^/SIRT1 axis downregulate Rab7 transcription and suppress RILP expression. As a result, impaired clearance of damaged LDs leads to excessive free fatty acid (FFA) release and aggravates myocardial injury (Ke et al., 2024).

#### 6.4.2 Golgi transport disorder

The Golgi apparatus, which orchestrates protein modification, sorting, and secretion, also contributes to lipid trafficking and metabolic equilibrium in cardiomyocytes. Under physiological conditions, it functions in tandem with the endoplasmic reticulum through COPI and COPII vesicles, facilitating bidirectional transport of proteins and lipids and maintaining the balance between lipid synthesis and degradation.

In DCM, persistent glucose and lipid overload renders the Golgi apparatus highly susceptible to lipid peroxidation. Elevated levels of 4-HNE covalently modify scaffold proteins such as GM130 and GRASP65, resulting in Golgi stack disassembly and fragmentation. This structural destabilization activates the CREB3–ARF4–mediated “Golgi stress” response, which upregulates pro-inflammatory and pro-apoptotic genes including CHOP, thereby exacerbating cellular injury. Golgi fragmentation also disrupts COPI/II vesicle formation, causing cytoplasmic retention of glucose transporter GLUT4 and fatty acid transporter CD36. This shifts substrate preference toward lipids over glucose, increasing reliance on β-oxidation and aggravating metabolic stress (van Gerwen et al., 2023).

#### 6.4.3 Peroxisome clearance disorders

Peroxisomes play a vital role in the β-oxidation of long-chain and very-long-chain fatty acids in cardiomyocytes. They house multiple oxidases that generate hydrogen peroxide (H_2_O_2_) as a byproduct, which is subsequently neutralized by catalase (Lismont et al., 2019). Under physiological conditions, peroxisomes work synergistically with mitochondria to degrade fatty acids and sustain intracellular redox balance (Okumoto et al., 2020). Unlike mitochondrial β-oxidation, the peroxisomal pathway consumes oxygen without producing ATP (Wanders and Waterham, 2006).

In DCM, marked by chronic high-glucose and high-fat exposure, cardiomyocytes divert excess long-chain and very-long-chain fatty acids into peroxisomes for β-oxidation as a compensatory “relief valve,” resulting in elevated H_2_O_2_ production. Covalent modification of catalase by 4-HNE, coupled with glyco-oxidative injury, impairs the peroxisome’s antioxidant defense, leading to redox disequilibrium. The resulting H_2_O_2_ overflow amplifies lipid peroxidation cascades. Due to their enrichment in polyunsaturated phospholipids, peroxisomal membranes are highly vulnerable to 4-HNE-induced oxidative damage, which inactivates membrane-associated proteins such as PMP70 and PEX5 and disrupts membrane integrity, giving rise to “leaky peroxisomes.” The uncontrolled release of ROS and matrix proteins into the cytosol intensifies oxidative stress and, through damaged mitochondria–peroxisome contact sites, impairs fatty acid trafficking and metabolic coordination, thereby aggravating lipotoxic injury in DCM.

## 7 Summary and prospects

In the development of DCM, 4-HNE, a key product of LPO, plays a pivotal pathogenic role. Within cardiomyocytes, 4-HNE induces coordinated damage to multiple organelles. However, research into 4-HNE and its associated signaling pathways remains limited by several challenges. First, although many mechanistic studies suggest a strong link between 4-HNE and DCM, the precise molecular targets and downstream pathways involved are not yet fully elucidated. Current models predominantly rely on STZ- or high-fat diet–induced mice, which fail to fully capture the pathological complexity of human T2DM. Broader validation across various experimental models and clinical populations is therefore necessary. Second, most identified 4-HNE targets have been observed only *in vitro* or in animal studies and lack systematic validation or quantitative profiling, limiting translational applicability.

Challenges also impede clinical translation and efficacy assessment of 4-HNE–targeted therapies in DCM. Orally administered agents undergo first-pass hepatic metabolism and extensive plasma protein binding, leaving limited effective exposure to the heart. Entry into—and residence within—key subcellular spaces (notably mitochondria) remain suboptimal. 4-HNE often rises in brief bursts; even slight timing mismatch between dosing and these peaks blunts efficacy. Scavengers or trapping molecules can react with non-target nucleophiles, introducing unexpected toxicity and narrowing the therapeutic window. Moreover, 4-HNE contributes to physiologic stress adaptation at baseline; excessive clearance risks disrupting necessary signaling. These limitations underscore the existing knowledge gaps and emphasize the need for continued in-depth research into 4-HNE–mediated mechanisms.
